# Ginger in patients with active ulcerative colitis: a study protocol for a randomized controlled trial

**DOI:** 10.1186/s13063-020-4193-7

**Published:** 2020-03-18

**Authors:** Forough Shayesteh, Fatemeh Haidari, Ali Akbar Shayesteh, Javad Mohammadi-Asl, Kambiz Ahmadi-Angali

**Affiliations:** 1 Department of Nutrition, Nutrition, and Metabolic Diseases Research Center, Ahvaz Jundishapur University of Medical Sciences, Ahvaz, Iran; 2 Alimentary Tract Research Center, Imam Khomeini Hospital, The School of Medicine, Ahvaz Jundishapur University of Medical Sciences, Ahvaz, Iran; 3 Department of Medical Genetics, Faculty of Medicine, Ahvaz Jundishapur University of Medical Sciences, Ahvaz, Iran; 4 Faculty of Public Health, Ahvaz Jundishapur University of Medical Sciences, Ahvaz, Iran

**Keywords:** Ulcerative colitis, Ginger, Inflammation

## Abstract

**Background:**

As a lifetime disorder, ulcerative colitis (UC) is an inflammatory bowel disease (IBD) that affects quality of life and also demands long-term interventions. In spite of considerable side effects and sometimes restricted uses, efficient medications are available for UC treatment. Some in vitro and in vivo examinations have correspondingly introduced ginger and its active components with antioxidant, anti-inflammatory, and anti-ulcerative properties. Therefore, this trial aims to evaluate the effect of ginger supplementation on patients with active UC.

**Methods:**

This study will be a 12-week, double-blind, parallel-group, randomized, controlled trial (RCT) in which 44 patients will be allocated to ginger and placebo groups receiving basic routine treatments plus ginger or placebo capsules, respectively. The primary outcomes are inflammatory markers (TNF-α and hs-CRP) and total antioxidant capacity.

**Discussion:**

The findings of this trial will provide evidence on the effect of ginger on patients with active UC.

**Trial registration:**

Iranian Registry of Clinical Trials, IRCT20190129042552N1. Registered on 21 June 2019.

## Background

Inflammatory bowel diseases (IBDs) are known as chronic recurrent disorders of the gastrointestinal tract and comprise two major types—ulcerative colitis (UC) and Crohn’s disease (CD)—that can result in poor quality of life and require long-term medical and/or surgical interventions [1]. The prevalence rates of UC have been reported as 37.5-248.6 and 4.9-505 per 100,000 individuals in Northern America and Europe, respectively [2]. Similarly, a systemic review also reported the prevalence rate of UC in Iran of 35.52 per 100,000 in the general population [3]. This disorder mainly develops in the rectum, and in some cases, it extends to the proximal colon, sometimes affecting the entire colon [1]. Moreover, its symptoms include diarrhea, abdominal pain, and fever, as well as clinical signs of intestinal obstruction and bleeding, depending on the location of the disease [1].

This inflammatory condition involves a wide range of alterations in inflammatory mediators such as tumor necrosis factor-α (TNF-α), interleukin-6 (IL-6), high-sensitivity C reactive protein (hs-CRP), and also a decrease in antioxidant capacity [4]. Furthermore, variations in microRNA (miRNA) expression have been reported in UC. MiRNAs are a group of single-strand, noncoding, RNA molecules that are 22 nucleotides long on average and regulate post-transcription gene expression [5].

MiR-21 is particularly relevant to intestinal inflammation in which overexpression has been reported in both patients and experimental models with IBD. MiR-21 play an important role in IBD pathogenesis (via gene expression regulation) [6], intestinal barrier function [7], intestinal microbiome [5], and the activation and differentiation of T cells [8, 9].

Pharmaceutical drugs for UC treatment include aminosalicylates (e.g., sulfasalazine and mesalazine), immunosuppressants (e.g., glucocorticoid, azathioprine, methotrexate, and cyclosporine A), and biologics (e.g., infliximab and adalimumab). Despite the efficacy of such drugs, some considerable side effects can limit their long-term use [10]. Therefore, the development of efficient, safe, and new therapeutic and complementary agents is necessary. In this respect, ginger, as an important agents, has been considered for its effects on chronic inflammation [11].

Ginger, viz. *Zingiber officinale* from the Zingiberacea family, is a rhizome widely used as a spice [12], which contains many active phenolic components such as gingerol, shogaol, and zingeron.

These components show antioxidative, anti-inflammatory, and immunomodulatory properties. A clinical trial evaluated the effect of ginger supplementation in older patients with osteoarthritis. This trial show a reduction in inflammatory markers like TNF-α and IL-1β [13]. In addition, another trial reported a reduction in nitric oxide and hs-CRP levels in ginger-supplemented osteoarthritis patients compared to the control group [14]. A randomized clinical trial conducted by Aryaeian et al. showed that ginger supplementation could improve the disease activity score and modulate some inflammatory genes in rheumatoid arthritis patients [15].

Data also have revealed that ginger suppresses nuclear factor kappa B (NF_κ_B) and ameliorates UC in experimental models [16, 17]. In a study by Allah et al., ginger administration (400 mg/kg) significantly improved the effects of acetic acid-induced colitis by decreasing the weight-to-length ratio of the colon and macroscopic and microscopic manifestation of colon specimens. These effects were associated with a significant decrease in NFkB expression and a decrease in TNF-α, IL-10, and the total peroxide levels in the colon. In this study, the therapeutic effects of ginger were shown to be more than preventive effects [16]. Dileep et al. investigated the anti-inflammatory effects of an alcohol extract of ginger (700 mg/kg/day) in ulcerative colitis induced by acetic acid in rats. The results showed that increased levels of carbonyl protein, TNF-α, IL-1β, and prostaglandin E2 decreased significantly in the colon. Significant increases in the levels of superoxide dismutase, catalase, reduced glutathione, glutathione peroxidase, and IL-10 were also observed in this study. Histological examination revealed a decrease in bleeding and edema in the colon of the treated group [18].

To the best of the authors’ knowledge, no published data exist on the effect of ginger supplementation on patients with UC; therefore, a randomized controlled trial (RCT) will be designed to determine the effect of ginger supplementation on the clinical status, serum levels of some inflammatory markers (i.e., TNF-α and hs-CRP), total antioxidant capacity (TAC), and expression of miR-21 in patients with active UC.

### Hypothesis and aim

The research hypothesis addressed is that ginger supplementation along with taking standard medications improves clinical and biochemical parameters. The primary objectives of this RCT is to assay the effect of 12-week ginger supplementation on inflammatory markers (TNF-α and hs-CRP) and TAC in patients with active UC. The secondary objective will be also to determine clinical score and miR-21 expression. For all outcomes, including the primary and secondary outcomes, measurements will be made at the beginning and the end of the intervention, and changes from baseline within groups and final values between groups will be evaluated.

## Methods/design

### Design and setting

**Fig. 1:**
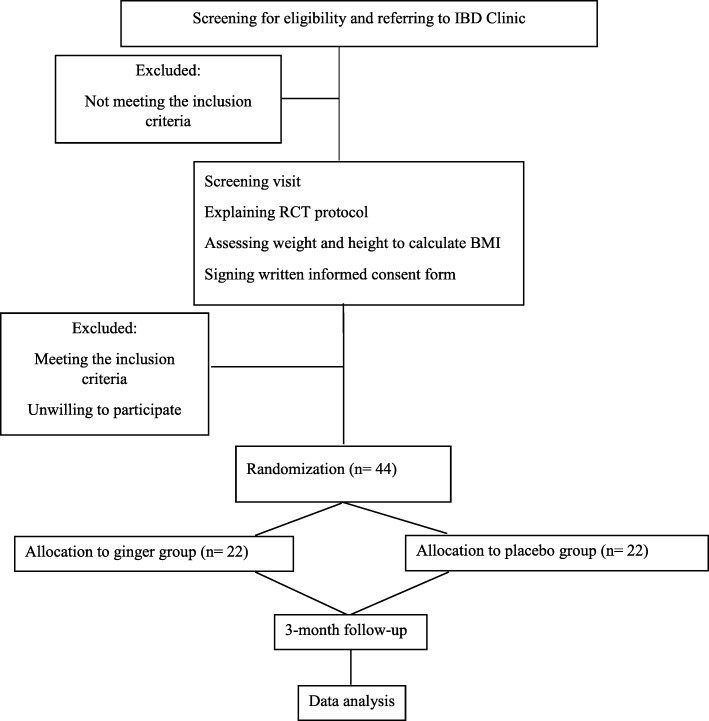
Overview of RCT protocol

A double-blind RCT with two parallel groups will be conducted. This RCT will conform to the Standard Protocol Items: Recommendations for Interventional Trials (SPIRIT) 2013 Statement (Additional file 1). The proposed clinical trial will be implemented in the IBD Clinic at Imam Khomeini Hospital, affiliated to Ahvaz Jundishapur University of Medical Science, Iran, for 12 weeks to assess the efficacy of daily adjunctive supplementation with 3 g ginger in patients with active UC. An overview of the study is illustrated in Fig. 1.

### Participants

The participants will be 44 patients with active UC. The inclusion criteria for eligibility of the participants will be as follows: moderate to severe active UC (according to the partial Mayo score; PMS); absence of fulminant UC signs; age range between 18 and 50 years; BMI between 18.5 and 30 kg/m^2^; absence of cancer or hepatic, renal, thyroid, and other gastrointestinal disorders; no sensitivity to ginger; no intake of nutritional supplements in the previous 8 weeks; and no involvement in other trials in the previous 12 weeks.

The exclusion criteria will be the following: use of anti-TNF-α drugs such as infliximab; pregnancy during the study; postmenopausal women; use of nonsteroidal anti-inflammatory and anti-diarrheal drugs; and use of anticoagulant, antidiabetic, or anti-pressure medications.

### Ethical considerations and trial registration

The Medical Ethics Committee of Ahvaz Jundishapur University of Medical Sciences approved this proposal, and it is in accordance with the Declaration of Helsinki (approval no.: IR.AJUMS.-REC.1397.893). Each participant will sign an informed written consent form before enrollment to this RCT. All collected data will be held confidential. The registration number for the Iranian Registry of Clinical Trials (IRCT registration no.) is IRCT20190129042552N1.

### Sample size

The sample size was calculated according to primary outcomes (if data were available), and the maximum calculated sample size was considered. The sample size was based on the changes observed in the TNF-α in the study by Mozafari Khosravi [13] as a result of ginger supplementation. Considering a type I error of 0.05, a test power of 90%, and follow-up loss of 10%, the sample size required in each group was calculated to be 22 patients. To achieve the estimated sample size, all patients will be included in the study in case they meet the inclusion criteria and are willing to participate in the study.

### Randomization and blinding

The patients will be randomly allocated to one of the intervention group (Group A) (standard treatment + ginger capsules, *n* = 22) or control group (Group B) (standard treatment + placebo capsules, *n* = 22). Random allocation sequence will be generated using a random number table by a third party who will be operationally independent from the study investigators and will generate a simple design. Ginger and placebo bottles will be also encoded based on random numbers by someone other than researchers, and odd or even numbers will be allocated randomly to Group A or Group B. To decrease the probability of bias, clinicians and those analyzing the results will be blind to the allocated groups. To maintain blinding, ginger and placebo bottles will be similar in appearance. Randomization codes of the study will remain confidential until all the patients have completed the study protocol.

### Intervention

During the 12-week intervention, both groups will continue the use of the prescribed medications. Patients in the intervention group will receive three ginger capsules on a daily basis (i.e., one capsule after each main meal), and each capsule will also contain 1 g of dried ginger rhizome. Notably, a human equivalent dose had been calculated in an animal study [14] using a conversion factor [19]. Patients in the control group will receive three placebo capsules daily; each capsule will contain 1 g of starch as a placebo, which looks like a ginger capsule in appearance. Ginger and placebo capsules are being made by the Green Plants Life Company. The method and the time of taking the capsules are labeled on the bottles. Patients in both groups will be requested not to change their typical diet and physical activities and also avoid the use of nutritional or ginger supplements, ginger candy, and ginger seasoning, as well as ginger tea and other herbal remedies during the study.

Ginger and placebo capsules will be delivered in identical containers for 4 weeks. The patients will be asked to give back the containers at the end of each fourth week for use in calculating their compliance rate. To ensure the ordered use of capsules as well as accurate implementation of the RCT, researchers will contact the patients via phone calls or short message service (SMS) each week.

### Measurements

At the onset of this RCT, demographic characteristics information will be collected via interviews and records, and relevant questionnaires will be completed. Weight will be also measured, while participants are wearing light clothes and no shoes, to 0.1-kg accuracy on a Seca scale, height will be measured, without shoes, using a 0.5-cm accuracy on a Seca stadiometer, and BMI will be calculated by dividing the weight (kg) by the height squared (m^2^).

In addition, the clinical status of the patients will be characterized by PMS and the Simple Clinical Colitis Activity Index (SCCAI) at pre- and post-intervention stages. Notably, the PMS is made up of three noninvasive parts of the complete Mayo score, which includes frequency of defecation, rectal bleeding, and a physician’s assessment of disease severity. Each of the parameters has a score from 0 to 3, and the maximum total score is 9. This score divides patients into those with inactive UC (i.e., in remission) and into those with active UC (i.e., mild, moderate, and severe). The clinical response is also defined as at least a 2-point reduction in the Mayo clinical score. The SCCAI includes the frequency of defecation during the night and day, defecation urgency, blood in stool, general sense of well-being, and external manifestations of the disease. The total score can range from 0 to 19.

Biochemical parameters consist of serum levels of TNF-α (ng/l), hs-CRP (mg/l), and TAC (u/ml) which will be evaluated by the enzyme-linked immunosorbent assay (ELISA) kits and miR-21 expression, which will be measured through reverse-transcription polymerase chain reaction (RT-PCR). At the beginning and the end of the study, a 5-ml venous blood sample (in a regular tube) will be collected in ethylenediaminetetraacetic acid (EDTA) tubes after approximately 12 h over-night fasting, and it will be centrifuged at 1500 g for 15 min and stored in −70 °C frigid temperature.

Physical activity will be evaluated by the short form of International Physical Activity Questionnaire (IPAQ) and calculated as MET-min/week. For dietary evaluation, 3 days of 24-h food recall (1 day of weekend and 2 days of the week) will be completed and then analyzed by Nutritionist IV software to determine the energy, protein, carbohydrate, and fat intake. Physical activity and dietary evaluation will be conducted at the onset and the end of the study.

### Analysis

The data will be analyzed using the Statistical Package for the Social Sciences (IBM SPSS Statistics 22. Ink). Normality of all quantitative variables (age, serum level of TNF-α, hs-CRP, TAC, and expression of mir-21) will be also evaluated by the Kolmogorov-Smirnov test. For normal distribution variables, independent sample t-test and paired sample t-test will be also used to compare parameters at the beginning and the end of the study between and within groups, respectively. The Mann-Whitney U test and Wilcoxon signed-rank test as nonparametric alternatives will be further applied to compare sample parameters between and within groups, respectively. In adjusted models for confounding variables (such as the type of medication), a general linear model (GLM) (univariate type) will be used for each certain parameter to compare mean values between groups. For each certain variable (primary and secondary outcomes), the percentage change will be also calculated (i.e., by subtracting the beginning from the final value, dividing by the beginning value, and multiplying by 100) and will be compared between groups by independent sample t-test or Mann-Whitney U, as well as by GLM univariate tests. The results will be presented as the mean ± SD, and differences will be considered statistically significant at *p* < 0.05. Intention-to-treat analysis will be employed if possible.

### Safety, adverse effects, and data monitoring

No side effects have been reported for 3 g/d consumption of ginger capsules [20]. However, any possible complications will be reported to the Medical Ethics Committee of Ahvaz Jundishapur University of Medical Sciences.

## Discussion

Use of alternative or complementary therapies (CAMs) has emerged as a common approach in gastrointestinal diseases. Therefore, different types of CAMs exist, and among them, herbal remedies seem much more appropriate for intestinal inflammation because of their primarily supposed safety and efficacy. Given their different active components, herbal remedies may simultaneously target multiple inflammatory response paths and mediators. However, most herbal remedies have an experimental basis, and proper experimentation on their safety and efficacy is needed to determine whether they can be considered as an appropriate strategy or not for improvement of IBD patients. Complications of medications also require an investigation into possible promising complementary agents. In this respect, ginger presents promising results in experimental models of UC. Therefore, this RCT will be designed to investigate the effect of ginger in patients with UC.

## Trial status

This trial is in the ongoing phase. The protocol version number is NCR-9722, and it was approved in March 2019. Patient recruitment began in July 2019 and is expected to be completed by May 2020.

## Supplementary information

**Additional file 1.** SPIRIT 2013 Checklist: Recommended items to address in a clinical trial protocol and related documents.

## Data Availability

The datasets generated and analyzed during the current study are not publicly available because this is a protocol, and the results are secure before publishing; however, these data are available from the corresponding author on reasonable request.

## Abbreviations

IBDs: Inflammatory bowel diseases
IPAQ: International Physical Activity Questionnaire
PMS: Partial Mayo score
UC: Ulcerative colitis
